# The effects of synbiotics supplementation on reducing chemotherapy-induced side effects in women with breast cancer: a randomized placebo-controlled double-blind clinical trial

**DOI:** 10.1186/s12906-023-04165-8

**Published:** 2023-09-26

**Authors:** Yasaman Khazaei, Ali Basi, Maria Luz Fernandez, Hossein Foudazi, Rafat Bagherzadeh, Farzad Shidfar

**Affiliations:** 1 Department of Nutrition, School of Public Health, Iran University of Medical Sciences, Tehran, 1449614535 Iran; 2 Department of Hematology Oncology, Iran University of Medical Sciences, Tehran, Iran; 3 Department of Nutritional Sciences, University of Connecticut, Storrs, CT USA; 4 Department of Radiation and Oncology, Shahid Fayaz-Bakhsh Hospital, Tehran, Iran; 5 English Department, School of Health Management and Information Sciences, Iran University of Medical Sciences, Tehran, Iran

**Keywords:** Chemotherapy-induced side Effects, Breast Cancer, Synbiotcs

## Abstract

**Background:**

The prevalence of breast cancer and its mortality rate are increasing rapidly among women worldwide. On other hand, the courses of chemotherapy as the main treatment for these patients are too much exhaustive and annoying. This study was designed to evaluate the use of synbiotics (probiotics + prebiotics) supplementation as a safe and inexpensive adjuvant treatment in reducing common chemotherapy side effects in women with breast cancer.

**Methods:**

The current study was conducted on 67 women with definitive diagnosis of breast cancer who were hospitalized to receive one-day chemotherapy sessions, and met the inclusion criteria. The patients were randomly allocated to the intervention or control group to receive synbiotics or placebo, respectively. They received oral consumption of synbiotics supplements twice a day for 8 weeks. The primary outcome was the changes in severity or experience of chemotherapy complication, analyzed by intention to treat (ITT). The instruments included 7 validated questionnaires which were used to assess chemotherapy complications in the initiation, 4 weeks and 8 weeks after intervention. Dietary intake was measured by 24-h dietary recall at the beginning, week 4 and week 8. Data were analyzed by SPSS software version 24. P-value < 0.05 was considered as statistically significant.

**Results:**

67 breast cancer patients participated in the study. 8 weeks after intervention and adjusting the confounders, the severity of chemotherapy complications including unnormal defecation (P = 0.005) and fatigue (P < 0/001) decreased significantly in the synbiotics group compared to the placebo group. Furthermore, nausea/vomiting (P = 0.015), and anorexia (P < 0.001) were decreased at the end of the study compared to the first visit, but it was not statistically significant compared to the placebo group.

**Conclusions:**

Synbiotics supplementation during chemotherapy can potentially reduce the severity of fatigue and abnormal defecation. It can help reduce anorexia and nausea/vomiting.

**Trial Registration:**

This study was registered in the Iranian Registry of Clinical Trials (IRCT) (registered code: IRCT20091114002709N56) (date of registration: 5/5/2021). Direct link to the trial page: https://www.irct.ir/trial/54559.

## Introduction

The most frequent malignancy among women all around the world is breast cancer( BC) with about 2 million diagnosed cases and 0.6 million deaths in 2020 [1, 2]. BC is the main cause of mortality in females aged 20–50 years and the second cause of cancer death in women after lung cancer [3]. The incidence rates of BC still are different across countries. There is a lower incidence and mortality in Asian regions, while in western countries more people die annually due to BC [4]. Although the exact etiology of BC is still unclear, a wide range of modifiable and nonmodifiable risk factors are known for the incidence of this malignancy, such as genetic features, ethnicity, family history, alcohol use, hormonal therapy, exercise, reproductive traits like pregnancy, and age of childbirth [5].

Chemotherapy is the main treatment in most patients with breast cancer. Cytotoxic agents damage and kill cancer cells through destroying DNA structure and stimulating apoptotic pathways; however, it can inevitably harm normal tissues, especially high proliferative ones, like the intestinal epithelial cells. Thus, chemotherapy often causes annoying side effects that may reduce the quality of a patient’s life [6, 7]. One of the direct detrimental effects of these agents is on the composition of normal intestinal microbiota defined as dysbiosis. This dysregulation may be an important cause of the occurrence of intestinal and/or non-intestinal complications [8]. Alternative therapies, like dietary or herbal supplements, additional vitamins and minerals, and recently live microorganisms have been considered by researchers for cancer management, i.e., preventing, treating, and reducing complications [9].

Anti-cancerous properties of probiotics, the live and beneficial microorganisms, marketed as food or supplements, have been frequently reviewed in previous studies. These properties can be reached by shifting the induced dysbiosis to eubiosis and homeostasis of microflora, and following advantages of normalizing cellular processes, metabolism, physiological pathways, and immunity [8, 10]. On another hand, the positive effects of probiotics on chemotherapy-induced side effects, and on gastrointestinal microbial homeostasis, especially in the microbiota- directly related cancers, like colorectal have been reported in recent studies [11–13].

Synbiotics, a combination of probiotic bacteria and polysaccharide as prebiotics, are less considered as adjuvant therapy in cancer cases, especially in cancers indirectly related to intestinal flora [14]. According to our literature review, this is the first clinical trial on the effects of synbiotics supplementation in reducing chemotherapy-induced side effects in breast cancer patients. Therefore, this double-blind placebo-controlled clinical trial aimed to evaluate the effect of oral intake of synbiotics supplements on common chemotherapy-induced complications, including nausea/vomiting, defecation problems, fatigue, pain, sleep quality, anorexia, and mental status in these patients.

## Materials and methods

### Study design

This double-blind randomized clinical trial was conducted in Firuzgar General Hospital, affiliated to Iran University of Medical Sciences, in Tehran, between April 2021 and April 2022. The project of this study was approved by the ethics committee of the Iran University of Medical Sciences (code number: IR.IUMS.REC.1400.050) and was registered in the Iranian Registry of Clinical Trials (IRCT) (registered code: IRCT20091114002709N56).

### Sample size

To calculate the sample size, according to the study design, Cochran’s sample size formula was used considering a type I error of 5% (α = 0.05) and SD power of 80% (β = 0.20) for all outcomes, such as pain, sleep quality, anorexia, and fatigue. Mean ± SD was calculated for the intervention and control groups for all variables. The largest calculated sample size was for fatigue. Considering fatigue as the primary outcome in this study and 20% dropout, a sample size of 37 participants was determined for each study group.

### Participants

The participants consisted of 74 inpatient women with a definitive diagnosis of breast cancer who were admitted to undergo neoadjuvant or adjuvant chemotherapy and met the inclusion criteria. The criteria for inclusion were 18 years of age or above with a recent definitive diagnosis of breast cancer by an oncologist or pathologist according to medical records with no metastasis, one-day chemotherapy session with at least one previous session to ensure that they had experienced chemotherapy-induced side effects following the first session, and at last four chemotherapy sessions with more than 2 future sessions after the follow-up visits. We selected the BC cases that were planned to get Adriamycin + Cyclophosphamide as chemotherapy agents, every 4 weeks. The exclusion criteria included:1) chronic diseases, such as liver disease (ALT and AST > 100 IU/L), kidney failure (serum creatinine > 1.7 mg/dl), or any abnormality in plasma cells counting (WBC > 20,000 U/L, hemoglobin < 10 mg/dl, platelets < 15,000 mc/L or > 400,000 mc/L), 2), previous gastrointestinal disease before cancer detection, 3) intake of probiotics or prebiotics or their supplements for two weeks before the study, 4) a history of tumor in other organs or any evidence of metastasis, 5) using medications or other treatments to reduce nausea, except the usual anti-nausea and vomiting medicines, 6) experiencing serious GI problems during the study, 7) a recent history of infection or taking antibiotics in the last three months, 8) reluctance to participate in the study. After the approval of the institutional review board, i.e., the educational supervisor of hospital, an informed consent was obtained from all patients.

**Fig. 1:**
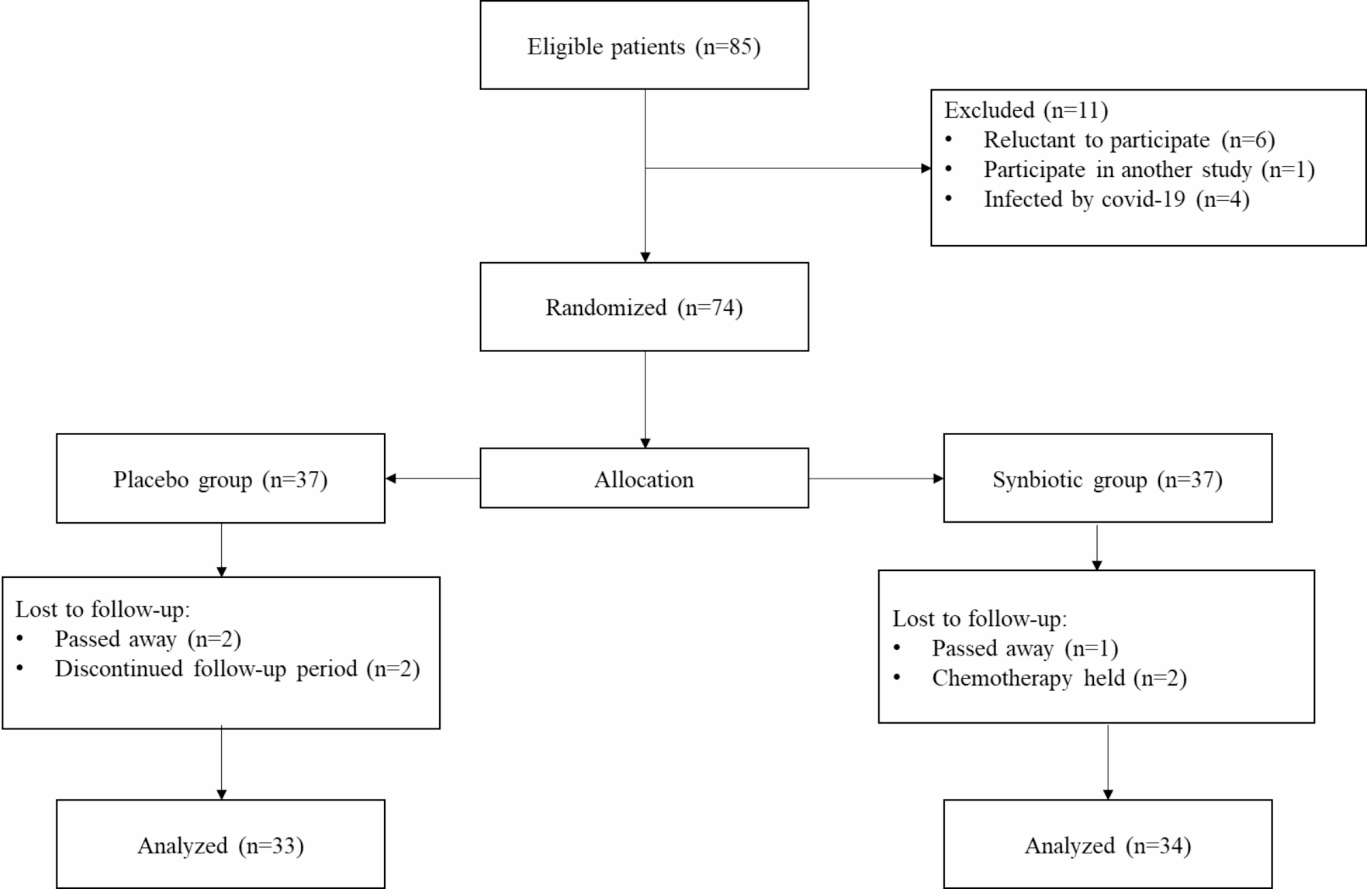
Consort diagram of study design

**Table 1:** Baseline characteristics of participants

Baseline characteristics			Groups			
			Synbiotics (n = 34)	Placebo (n = 33)	P value	
Age (years)			53.58 ± 11.34	51.00 ± 12.53	0.390^2^*	
Education	illiterate		1 (%2.9)	0 (%0 )	0.079^1^	
	elementary		12 (%35.3 )	9 (%27.3)		
	High school		18 (%52.9)	13 (%39.4)		
	university		3 (%8.8)	11 (%33.3)		
Disease history	none		30 (%88.2)	27 (%81.8)	0.414^1^	
	diabetes		2 (%5.8)	5 (%15.2)		
	dyslipidemia		2 (%5.9)	1 (%3)		
Chemotherpy type	adjuvant		17 (%50)	19 (%57.6)	0.534^1^	
	Neuadjuvant		17 (%50)	14 (%42.4)		
Occupation	Non-employee		25 (%73.5)	23 (%69.7)	0.728^1^	
	Employee		9 (%26.5)	10 (%30.3)		
Smoking	yes		8 (%23.5)	5 (%15.2)	0.386^1^	
	No		26 (%76.5)	28 (%84.8)		
Marriage	Single		5 (%14.7)	7 (%2.21)	0.478^1^	
	Married		29 (%85.3)	26 (%78.8)		
Tumor Surgery	yes		17 (%50)	19 (%57.6)	0.534^1^	
	No		17 (%50)	14 (%42.4)		
	None		13 (%38.2)	9 (%27.2)		
Supplements intake	Multivitamin		10 (%29.4)	8 (%24.2)	0.329^1^	
	Vitamin D		19 (%55.8)	20 (%60.6)		
	Calcuim		6 (%17.6)	9 (%27.2)		
Medication (except for common medicines)	None		30 (%88.2)	27 (%81.8)	0.414^1^	
	Diabetes		2 (%5.9)	5 (%15.2)		
	Dyslipidemia		2 (%5.9)	(%3)		
Chemotherapy session	Second		22 (%64.7)	20 (%60.6)	0.349^1^	
	Third		8 (%23.5)	5 (%15.2)		
	Forth		4 (%11.8)	8 (%24.2)		

Quantitative data reported as mean ± SD, and qualitative data reported as number (%)

^1^Between-group analysis conducted via chi-square test

^2^Parametric and non-parametric quantitative variables assessed via Independent-Samples T test ^¥^ or Mann-Whitney*, respectively

### Randomization and masking

A trained interviewer (a qualified dietitian) was asked to stratify the patients into four random blocks: A, B, C and D, based on their matched characteristics, (age: 18–40, and 40–70 years old, body mass index (BMI): 18.5–24.9, and 25–25,9). In the next step a statistical software was used to randomly allocate the patients in each block to either an intervention group or a control group. Randomization and masking were done in order to maintain allocation concealment. The participants and researchers were masked to the type of the supplements they received; only the coordinator of company of supplements was aware of the type of the supplements and he was required to keep the information until the end of the analysis. If deviation from the prescribed protocol had occurred, the data would have been analyzed by intention-to-treat (ITT) method; therefore, data analysis at the end of the study was performed with due consideration to randomization that was done at the beginning of the study.

**Table 2:** Physical activity of participants at baseline, week 4, and week 8

	Baseline		Week 4		Week 8		
Physical activity severity	Synbiotics (n = 34)	Placebo (n = 33)	Synbiotics (n = 34)	Placebo (n = 33)	Synbiotics (n = 34)	Placebo (n = 33)	
Mild	28 (%82.4)	29 (%87.9)	29 (%85.3)	29 (%87.9)	31 (%91.2)	31 (%93.9)	
Moderate	6 (%17.6)	4 (%12.1)	5 (%14.7)	4 (%12.1)	3 (%8.8)	2 (%6.1)	
Severe	0 (%0)	0 (%0)	0 (%0)	0 (%0)	0 (%0)	0 (%0)	
P value^1^	0.526		0.756		0.667		

Data reported as number (%)

^1^Between-group comparison conducted via Chi-square test.

### Study protocol

The details of the study were explained to every single patient and their companions. After obtaining informed consent from the patients, we filtered them based on our inclusion and exclusion criteria, and eligible participants were randomized to receive placebo or synbiotics supplements. During the first visit, the trained dietitian distributed a demographic questionnaire among the patients to collect basic information, such as age, marital status, smoking status, education, history of surgery, date of cancer diagnosis, the sessions of chemotherapy, medicine or supplement use, and chronic or acute disease history. Then, as the pre-test, seven validated questionnaires on experiences of chemotherapy-induced side effects were distributed among the patients by the same individual. The reliability and validity of the questionnaires were approved in previous study on cancer patients. Moreover, the International Physical Activity Questionnaire (IPAQ) was completed by the researchers to check the patients’ physical activity levels, based on metabolic equivalent-h/d (MET-h/d), and a 24-h dietary recall to assess dietary intakes. After the pre-test, each woman received 56 capsules of synbiotics or placebo. They were requested to take one capsule twice a day, after their main meals, and to store them in the refrigerator below 4 °C. Four weeks later, in the next chemotherapy session, the questionnaires on experiences of chemotherapy-induced side effects were given to the patients for the second time, and anthropometric measurement was repeated. Then, 56 more capsules were given to the patients to take for the next four weeks, until the last visit. In the third visit, all procedures in previous sessions were repeated.

### Characteristics of synbiotics and placebo

The synbiotics supplement and placebo capsules were identical (Lactocare®) and were made by ZistTakhmir Company, Tehran, Iran. Each capsule weighed 450 mg and contained 12 safe and useful probiotic strains (*Lactobacillus rhamnosus, Lactobacillus casei, Lactobacillus acidophilus, Lactobacillus bulgaricus, Bifidobacterium breve, Bifidobacterium longum, Lactobacillus helveticus, Lactobacillus lactis, Lactobacillus paraplantarum, Bifidobacterium bifidum, Streptococcus thermophilus and Lactobacillus gasseri*) with a dose of 1 × 10^9^ CFU, and 21 g fructooligosaccharides as prebiotic. The placebo contained starch, and was similar to the supplement in color, odor, and shape. Supplement and placebo were packed in identical packages and labeled as either A or B by the company assistant to guarantee blinding. It was a double-blind study, both the patients and investigators were unaware of the administered product.

**Table 3:** Anthropometric characteristics of participants

		Groups		
Anthropometric variables		Synbiotics (n = 34)	Placebo (n = 33)	**P value**
Height (cm)		161.20 ± 4.23	161.12 ± 5.21	0.942^¥^
Weight (kg)	baseline	64/16 ± 6.46	65.69 ± 7.75	0.517*
	Week 4	64.47 ± 6.23	65.52 ± 8/07	0.692*
	Week 8	64.62 ± 6.09	65.19 ± 8.43	0.750*
BMI (kg/m^2^)	baseline	24.62 ± 2.45	25.25 ± 2.48	0.301*
	Week 4	24.76 ± 2.37	25.19 ± 2.62	0.603*
	Week 8	24.82 ± 2.24	25.12 ± 2.63	0.783*
Waist circumference (cm)	baseline	86.79 ± 9.60	87.15 ± 10.62	0.886^¥^
	Week 4	86.97 ± 9.42	86.90 ± 10.83	0.980^¥^
	Week 8	87.26 ± 9.17	86.84 ± 10.88	0.886^¥^
Data reported as number (%) ^1^Between-group comparison conducted via Chi-square test.				

data reported as mean ± SD

Repeated Measures ANOVA was used to analysis of time-effect^1^, group-effect^2^, and time-group-effect^3^ between-groups comparison conducted via Repeated Measures ANOVA

### Anthropometric assessments

All patients were weighed in light clothes and barefoot on Seca digital scale with an accuracy of 0.5 kg. Moreover, they were asked to stand straight back and heels against the wall and look straight forward, then a thin non-elastic tape meter was used to measure their height to the nearest 0.1 cm [15].BMI was calculated as body weight (kg) divided by height in meters squared (m^2^). The International Physical Activity Questionnaire (IPAQ) was used to assess the physical activity level, based on metabolic equivalent-h/d (MET-h/d) [16]. In order to measure waist circumference (cm), the patients were asked to stand upright and breathe normally, then the right ilium of the pelvis was located, and a thin non-elastic tape-meter, parallel to the floor, was used to measure waist circumference with a 0.1 cm level of precision.

### Dietary intake and physical activity assessments

The dietitian assessed dietary intakes of participants in each visit by using 24-h dietary recall at baseline to 4 and 8 weeks after intervention. Data were analyzed by Nutritionist IV software (First Databank, San Bruno, Calif, USA) modified for Iranian food. The physical activity of participants was measured by using the International Physical Activity Questionnaire (IPAQ) at baseline to 4 and 8 weeks after intervention.

### Questionnaires of outcome assessment

#### Defecation quality assessment: Bristol stool form scale

Stool consistency was assessed according to the Bristol Stool Scale adopted from Lewis et al. [17], a visual scale that classifies feces into seven categories according to the thickness and colonic transit time. This scale represents a spectrum with scores ranging from 1 to 7, where 1 equals very dried feces, while 7 means completely liquid feces. Scores 3, 4, and 5 are considered as normal feces. During each visit, a printed picture of this categorization was shown to the patients, and they determined their predominant stool pattern within the previous four weeks.

**Table 4:** Dietary intakes of participants at baseline, week 4, and week 8.

		Groups				
Energy and nutrients intake		Synbiotics (n = 34)	Placebo (n = 33)	^1^P value	^2^P value	^3^P value
Energy (Kcal/d)	Baseline	1538.81 ± 436.68	1694.89 ± 483.68	0.208	0.163	0.306
	Week 4	1704.93 ± 412.79	1491.58 ± 483.68			
	Week 8	1740.15 ± 431.56	1420.33 ± 527.00			
	P value^4^	< 0.001	< 0.001			
Carbohydrate (gr/d)	Baseline	210.73 ± 58.64	216.19 ± 73.09	0.524	0.623	0.238
	Week 4	228.10 ± 58.60	204.32 ± 68.73			
	Week 8	231.87 ± 61.71	194.04 ± 65.01			
	P value^4^	0.068	0.072			
Protein (gr/d)	Baseline	68.49 ± 13.99	61.97 ± 19.91	0.015	0.004	0.003
	Week 4	70.77 ± 14.20	59.91 ± 18.89			
	Week 8	80.53 ± 14.21	62.18 ± 21.25			
	P value^4^	< 0.001	0.099			
Fat (gr/d)	Baseline	63.10 ± 17.31	64.76 ± 19.53	0.059	0.041	0.064
	Week 4	68.44 ± 20.90	54.40 ± 20.63			
	Week 8	67.40 ± 23.73	52.66 ± 21.19			
	P value^4^	0.060	< 0.001			
Fiber (g/d)	Baseline	38.91 ± 17.77	36.44 ± 10.56	0.060	0.023	0.353
	Week 4	38.20 ± 18.22	35.36 ± 8.66			
	Week 8	38.22 ± 18.41	33.59 ± 9.19			
	P value^4^	0.628	0.013			
Saturated fatty acids (g/d)	Baseline	15.62 ± 4.61	15.78 ± 3.59	0.012	0.656	0.935
	Week 4	15.39 ± 3.80	15.24 ± 3.09			
	Week 8	15.31 ± 3.87	15.51 ± 2.43			
	P value^4^	0.748	0.094			
Poly unsaturated fatty acids (g/d)	Baseline	10.15 ± 5.92	11.95 ± 5.78	0.855	0.861	0.859
	Week 4	10.97 ± 6.20	11.13 ± 6.78			
	Week 8	11.69 ± 6.03	10.55 ± 6.22			
	P value^4^	0.548	0.081			
Mono unsaturated fatty acids (g/d)	Baseline	28.66 ± 10.58	31.03 ± 10.35	0.571	0.391	0.638
	Week 4	29.39 ± 10.75	30.82 ± 10.93			
	Week 8	30.13 ± 10.88	30.15 ± 10.83			
	P value^4^	0.891	0.488			
Trans fatty acids (g/d)	Baseline	0	0	1.000	1.000	1.000
	Week 4	0	0			
	Week 8	0	0			
	P value^4^	1.000	1.000			
cholesterol (g/d)	Baseline	204.33 ± 57.50	206.38 ± 62.10	0.080	0.814	0.668
	Week 4	203.77 ± 56.33	211.01 ± 55.24			
	Week 8	186.64 ± 60.82	193.85 ± 52.38			
	P value^4^	0.061	0.073			
Iron (mg/d)	Baseline	12.34 ± 1.58	12.50 ± 2.21	0.296	0.140	0.965
	Week 4	12.57 ± 1.59	12.78 ± 1.50			
	Week 8	12.79 ± 1.31	12.39 ± 1.33			
	P value^4^	0.051	0.062			
Magnesium (mg/d)	Baseline	310.50 ± 69.96	305.81 ± 80.48	0.132	0.410	0.412
	Week 4	316.43 ± 83.57	295.46 ± 83.81			
	Week 8	301.74 ± 77.98	282.33 ± 83.84			
	P value^4^	0.337	0.062			
Zinc (mg/d)	Baseline	6.91 ± 1.08	8.03 ± 1.53	0.025	0.101	0.745
	Week 4	7.23 ± 1.05	7.55 ± 1.34			
	Week 8	7.35 ± 1.05	7.35 ± 1.37			
	P value^4^	0.074	0.082			
Calcium (mg/d)	Baseline	568.44 ± 262.85	678.26 ± 239.02	< 0.001	< 0.001	0.079
	Week 4	611.06 ± 282.22	614.42 ± 264.21			
	Week 8	765.52 ± 279.64	572.39 ± 228.17			
	P value^4^	< 0.001	0.001			
Vitamin D (µg/d)	Baseline	1.36 ± 1.32	1.85 ± 1.68	0.385	0.103	0.741
	Week 4	1.33 ± 1.29	1.62 ± 1.56			
	Week 8	2.58 ± 5.50	1.35 ± 1.52			
	P value^4^	0.428	0.092			
Vitamin C (mg/d)	Baseline	71.18 ± 23.32	56.19 ± 28.20	< 0.001	< 0.001	< 0.001
	Week 4	77.03 ± 25.88	55.33 ± 25.53			
	Week 8	84.49 ± 27.81	53.94 ± 26.49			
	P value^4^	< 0.001	0.287			
Copper (mg/d)	Baseline	1.26 ± 0.35	1.19 ± 0.36	0.070	0.863	0.233
	Week 4	1.16 ± 0.28	1.06 ± 0.23			
	Week 8	1.21 ± 0.32	1.13 ± 0.45			
	P value^4^	0.165	0.115			

data reported as mean ± SD

Repeated Measures ANOVA was used to analysis of time-effect^1^, group-effect^2^, and time-group-effect^3^ between-groups comparison conducted via Repeated Measures ANOVA

#### Nausea and vomiting assessment: rhodes index of nausea and vomiting -form2 (INV2) index

Rhodes IVN2 index, developed by Rhodes et al. in 1986 [18], is a valid measurement tool for. assessing three subscales, including (1) frequency, distress, and amount of nausea, (2) frequency, distress, and amount of vomiting, and (3) frequency and distress of retching. It consists of eight multiple-choice questions. The scoring and interpretation are based on the Likert rating. Each question could get between 0 and 4 scores. Accordingly, we added the scores of each question together, to calculate the total score ranging from 0 to 32. A higher score means more nausea and vomiting, and vice versa.

#### Fatigue assessment: cancer fatigue scale (CFS Questionnaire)

To assess fatigue in participants, we used the Persian version of the Cancer Fatigue Scale Questionnaire (CFS), the reliability and validity of which were approved previously [19, 20]. This concise questionnaire comprises 15 Likert-type items and 3 subscales assessing physical, affective, and cognitive aspects of fatigue. The ratings are assigned the values 1 to 5 (where 1 = not at all, 2 = a little, 3 = somewhat, 4 = a lot, and 5 = very much) [21, 22]. The scores of each item were marked by the interviewer according to the patient’s response. The total score of the questionnaire ranged from 0 to 60 points. The higher score reflects more fatigue.

#### Pain assessment: McGILL pain questionnaire (MPQ)

The experience and severity of pain among patients were measured by using the Persian version of McGILL pain questionnaire (MPQ) the reliability and validity of which were approved in a study by Khosravani et al. on cancer patients in Iran [23]. It consists of 78 pain-descriptive words arranged into 20-word collections. The patients could choose only one word from each collection. If 2 words were selected for describing their pain, the word with higher score was considered. The total score of MPQ is calculated by the sum of the scores of all collections. The total score ranges 0–78 scores (0 means no word is chosen, and 78 means the last description in all collections is chosen. A higher score reflected more severe pain [24].

**Table 5:** Within- and between-group comparison of the changes in chemotherapy-induces complications during and after intervention

Complications	Synbiotics (n = 34)					Placebo (n = 33)					std. Error	B	^6^Adjusted P value	^5^Unadjusted P value
	Baseline	Week 4	Week 8	^1^P value _1,2_	^2^P value _1,3_	Baseline	Week 4	Week 8	^3^P value_1,2_ 1,2	^4^P value_1,3_				
**Nausea/vomiting**	20.35 ± 9.07	18.35 ± 8.39	17.17 ± 8.80	0.266	0.015	20.69 ± 5.22	21.57 ± 4.41	22.06 ± 4.54	0.450	0.386	1.867	1.591	0.394	0.085
**Fatigue**	40.29 ± 4.29	33.85 ± 4.82	30.55 ± 4.95	< 0.001	< 0.001	39.09 ± 4.54	39.72 ± 4.97	40.69 ± 5.09	0.195	0.032	1.373	5.069	< 0.001	< 0.001
**Pain**	4.17 ± 8.76	3.91 ± 7.17	3.82 ± 6.99	0.217	0.163	2.75 ± 4.18	2.84 ± 4.12	2.72 ± 3.94	0.734	0.887	1.252	− 0.710	0.570	0.414
**Anorexia**	20.61 ± 5.45	26.11 ± 7.02	28.64 ± 9.74	< 0.001	< 0.001	22.58 ± 5.88	22.60 ± 5.64	22.66 ± 5.88	0.910	0.624	1.731	− 0.934	0.590	0.097
**Anorexia (VAS)**	4.79 ± 1.43	5.85 ± 0.98	6.47 ± 1.61	< 0.001	< 0.001	4.96 ± 1.51	5.03 ± 1.48	4.87 ± 1.55	0.070	0.067	0.331	− 0.138	0.678	0.015
**Sleep quality**	12.88 ± 5.15	12.55 ± 4.78	12.35 ± 4.99	0.902	0.824	12.66 ± 4.45	12.57 ± 4.17	12.87 ± 4.37	0.790	0.901	1.234	− 0.049	0.968	0.921
**Mental status**	16.20 ± 8.21	15.02 ± 8.38	14.85 ± 8.29	0.156	0.533	16.06 ± 8.30	16.36 ± 8.52	16.24 ± 8.49	0.374	0.222	2.257	− 2.026	0.370	0.668
**Weight (kg)**	64.16 ± 6.56	64.47 ± 6.23	64.62 ± 6.09	0.825	0.574	65.69 ± 7.75	65.52 ± 8.09	65.19 ± 8.43	0.283	0.148	1.851	1.553	0.402	0.545
**BMI (kg.m** ^**2**^ **)**	24.62 ± 2.45	24.76 ± 2.37	24.82 ± 2.24	0.559	0.299	25.25 ± 2.48	25.19 ± 2.62	25.12 ± 2.63	0.168	0.218	0.523	0.577	0.271	0.443
**Waist circumference (cm)**	86.79 ± 9.60	86.97 ± 9.42	87.26 ± 9.17	0.901	0.631	87.15 ± 10.62	86.90 ± 10.86	86.84 ± 10.88	0.643	0.537	2.384	1.144	0.631	0.987

data reported as mean ± SD

^1^within-group comparison between first and second visits in the synbiotics group conducted via GEE analysis (adjusted for energy, protein, calcium, and vitamin C intake)

^2^within-group comparison between second and third visits in the synbiotics group conducted via GEE analysis (adjusted for energy, protein, calcium, and vitamin C intake)

^3^ within-group comparison between first and second visits in the placebo group conducted via GEE analysis (adjusted for energy, fat, calcium, and fiber intake)

^4^within-group comparison between second and third visits in the placebo group conducted via GEE analysis (adjusted for energy, fat, calcium, and fiber intake)

^5^between-groups comparison conducted via GEE analysis

^6^between-groups comparison conducted via GEE analysis (adjusted for protein, fat, fiber, calcium, and vitamin C intake)

#### Anorexia assessment: functional assessment of anorexia and cachexia therapy (FAACT) questionnaire- anorexia cachexia subscale (AC/S) -12 part

To assess anorexia and appetite status of the patients, we used AC/S-12 that is the fourth part of FAACT, a validated questionnaire to evaluate the quality of life in cancer patients. AC/S-12 subscale has been frequently performed in previous studies to assess anorexia and cachexia in cancer patients. It’s validity and reliability have been previously confirmed [25]. These subscales contain 12-items ranging from 0 to 4 scores (0 = not at all, 1 = a little bit, 2 = somewhat, 3 = quite a bit, and 4 = a lot). The total score ranges from 0 to 48 that is the sum of scores of all 12 items. Higher score represents a better appetite [26].

#### Sleep quality assessment: persian version of the Pittsburgh sleep quality index (PSQI)

To assess the sleep quality of patients, the Persian version of the Pittsburgh Sleep Quality Index (PSQI), designed by Buysse et al. in 1989 [27], was used. The reliability and validity of the Persian version of PSQI have been confirmed by Farrahi Moghaddam et al. who examined its sensitivity (100%,) specificity(93%), and Cronbach’s alpha (0.89% ) [28]. The questionnaire includes 19-items on seven features of sleeping quality, latency, duration, efficiency, disturbances, medication use, and daytime problems. Each item is rated on a four-point scale (0–3) where 0 indicates a high quality of sleeping, and 3 represents a poor sleep quality. The total score of PSQI ranges from 0 to 21. A higher score is indicative of worse sleep quality [29].

#### Mental status assessment: PHQ-9 questionnaire

To assess the mental status of the patients, we employed the Persian version of Patient Health Questionnaire-9 (PHQ-9) the validity and reliability of which were approved by Dadfar et al. in 2018 [30]. This self-reported questionnaire is made up of 9 items evaluating the existence and severity of anxiety and depression over the past 2 weeks, based on the Diagnostic and Statistical Manual of Mental Disorders (DSM-IV) criteria. Each item is rated on a four-point Likert scale (0: not at all, 1: some days, 2: more than half of the days, 3: almost every day). The total score ranges from 0 to 27. The severity cut-points include < 5 (none), 5–9 (mild), 10–14 (moderate), 15–19 (almost severe), and > 19 (severe). In terms of quantity and comparison, scores closer to 27 imply the severity of the problem [31, 32].

#### Safety of assessment

All participants were in contact with the researcher from the beginning to the end of the follow-up visits via telephone. They were asked to report any experience of unexpected or critical symptoms and signs.

### Statistical analysis

Participants were compared on different baseline characteristics; therefore Independent-sample t-test and Mann-Whitney U-test analyses were employed based on normal and non-normal distribution, respectively. Normality of variables was assessed by Shapiro-wilk test. The comparison of qualitative variables was performed by using Chi square analysis. The quantitative and qualitative data were represented as mean ± SD and numbers (%), respectively. Repeated measures ANOVA was performed to determine the changes of dietary intake of the participants during the study (time effect), the difference between placebo and synbiotics intake (group effect), and the interaction of time and group on changes of participants’ dietary intake (time-group effect). To determine the effects of synbiotics supplementation on each chemotherapy-induces side effect, Generalized Estimating Equations (GEE) model was used after transforming big data to longitudinal data. All analyses were performed by Statistical Package for the Social Sciences (SPSS) version 23.0 (Chicago, IL, USA). P value < 0.05 was considered statistically significant.

## Results

Between April 2021 and April 2022 and after the assessment of 85 primary eligible patients, 74 patients were randomly allocated to synbiotics and placebo groups (Fig. 1). Some patients were initially excluded (n = 11) since they were reluctant to participate in the study (n = 6), were on another intervention (n = 1), or were infected by COVID-19 (n = 4). Also, during the follow-up period, we lost 3 patients in the synbiotics group due to death (n = 1) and chemotherapy (n = 2); similarly, we lost 4 patients in the placebo group due to death (n = 2) and reluctance to continue the study (n = 2). Ultimately, the final analysis was performed on 34 patients in the synbiotics and 33 patients in the placebo groups.

The homogeneity of the baseline features was shown in two groups (Table 1). The mean ages were 53.58 ± 11.34 and 51.50 ± 12.53 years in the synbiotics and control groups, respectively. There were no significant differences between the study groups in assessed demographic characteristics (age, education, history of chronic disease, chemotherapy type, occupation, smoking status, marriage status, surgery status, chemotherapy session, supplements or medicines usage) at baseline (Table 1).

Furthermore, there were no significant differences in physical activity levels (Table 2) and anthropometric measurements (weight, height, BMI, and waist circumference) between the two groups at the beginning and also at the end of the study (Table 3). No significant difference was reported between the two groups at the end of study, within group changes were not significant either. There was no evidence of adverse or unexpected effects following supplements or placebo consumption during the entire time of intervention. The energy, micro- and macronutrients intake of the two groups are shown in Table 4, separately, in three times of assessments, i.e., at baseline 4 weeks and 8 weeks after intervention. In addition, there were no significant differences between the two groups in their dietary intake, except for protein (P = 0.004), fat (P = 0.041), fiber (P = 0.023), calcium (P < 0.001) and vitamin C (P < 0.001). The rest of the nutritional intakes were homogenous. At the end of the study, compared to the baseline, in the synbiotics group the intake of energy (P < 0.001), protein (P < 0.001), calcium (P < 0.001) and vitamin C (P < 0.001) increased, whereas in the placebo group the intake of energy (P < 0.001), fat (P < 0.001), fiber (P = 0.013) and calcium (P = 0.001) decreased (Table 4).

The total scores of each chemotherapy-induced side effects are reported in Table 5 at baseline, 4 weeks and 8 weeks after intervention for the synbiotics and placebo groups.

**Table 6:** The assessment of defecation quality of participants

**Group**													
**Variable**			Synbiotics (n = 34)			Placebo (n = 33)			**B**	**Upper** ^**3**^	**Lower** ^**2**^	**OR** ^**4**^	**P value** ^**1**^
			Baseline	Week 4	Week 8	Baseline	Week 4	Week 8					
**Defecation**		**Abnormal**	32 (%94.1)	1 (%2.9)	1 (%2.9)	32 (%97)	30 (%90.9)	29 (%87.8)	1.392	67.22	12.35	15.618	0.005
		**Normal**	2 (%5.9)	33 (%97.1)	33 (%97.1)	1 (%3)	3 (9.0%)	4 (%12.2)					

data reported as mean ± SD

^1^Between-groups comparison conducted via GEE analysis (adjusted for protein, fat, fiber, calcium and vitamin C intakes)

^2^ Lower limit of confidence interval

^3^Upper limit of confidence interval

^4^Odds Ratio

**Fig. 2:**
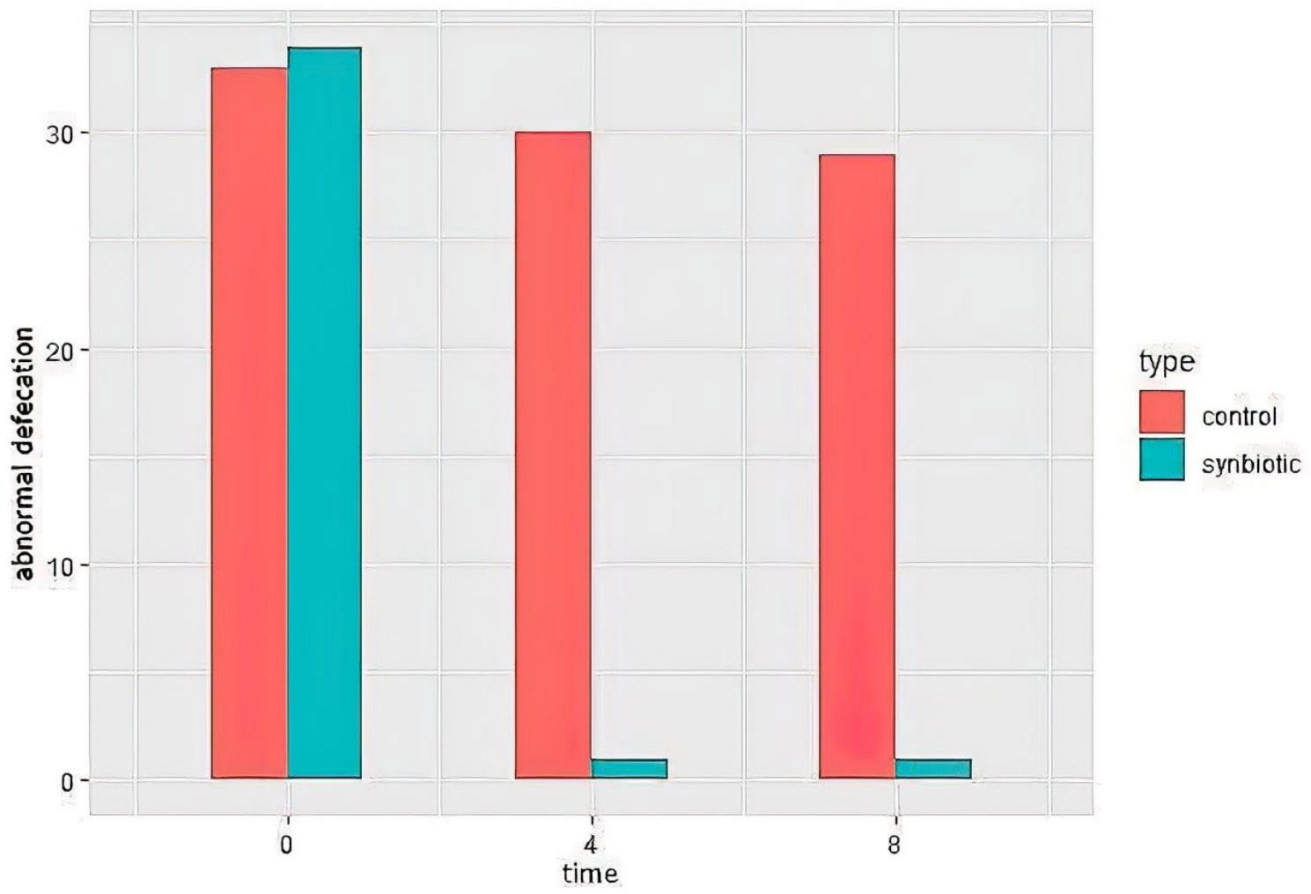
Changes of defecation quality weight in synbiotics and control groups during the study. Higher score means worst defecation quality

Based on the visual Bristol scale, we observed a clear significant improvement in defecation quality of patients at the end of study in the synbiotics group compared to that of the placebo group (P = 0.005) (Fig. 2). According to the GEE analysis, taking synbiotics could potentially ameliorate the defecation problems in these patients for about 15 time more than placebo (OR: 15.61; 95% CI: (12.35–67.22)). As presented in Tables 6 and 8 weeks after taking synbiotics supplementation, there was a 100 reduction in the incidence of diarrhea among patients with diarrhea in the synbiotics group (100% improvement). This efficient effect was observed after 4 weeks, too. Whilst, in the placebo group, after 8 weeks, 18 out of 20 patients still reported diarrhea, (15% improvement). In addition, only 1 out of 14 patients with constipation in the synbiotics group reported this abnormal defecation 4 and 8 weeks after intervention (92.8% improvement). While, all 12 patients with constipation in the placebo group, still had the same problem at the end of the study (0% improvement).

**Fig. 3:**
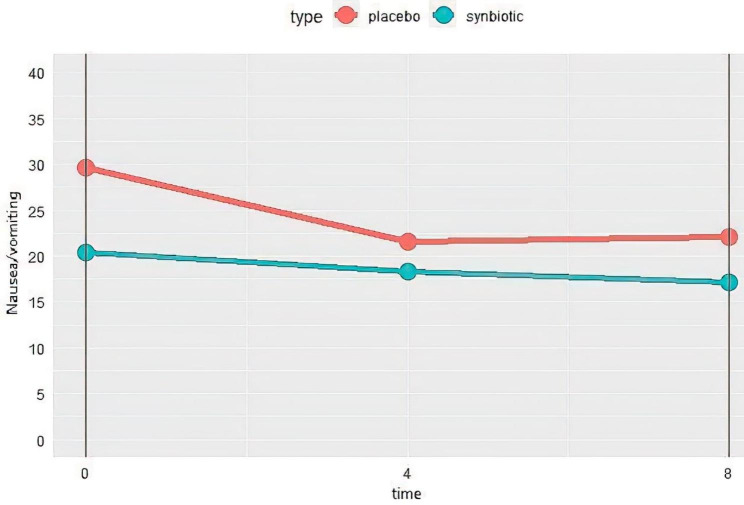
Changes of nausea/vomiting severity in synbiotics and control groups during the study. Higher score means more nausea/vomiting

The changes of nausea/vomiting complication are shown in Fig. 3. As it is shown in the table, 8 weeks after the consumption of synbiotics supplements, the severity of nausea/vomiting significantly decreased in the synbiotics group, compared to the first visit (P = 0.015); however, no significant difference was observed at week 4. Although nausea/vomiting experience increased in the placebo group during the study, it was not statistically significant. In general, there was no statistically significant difference between the two groups (Table 5).

**Fig. 4:**
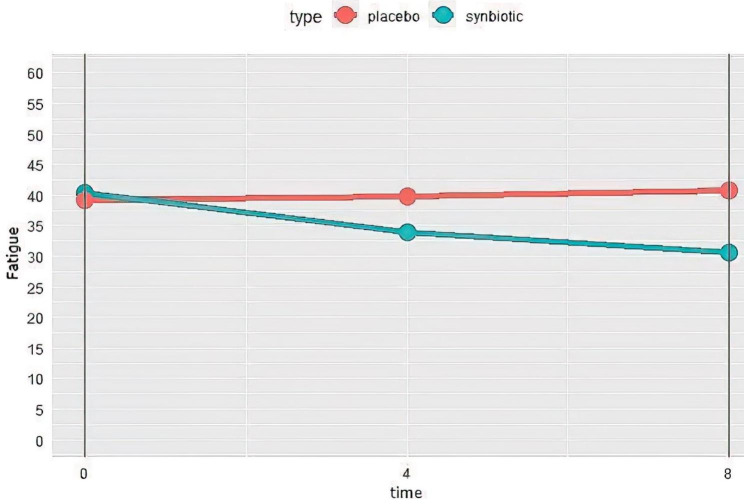
Changes of fatigue severity in synbiotics and control groups during the study. higher score means more fatigue

A significant reduction was reported in fatigue severity in the synbiotics group after 8 weeks, compared to the first visit (P < 0.001). This significant reduction was also observed at week 4 (P < 0.001). Nevertheless, the severity of fatigue increased in the placebo group (P = 0.032) after 8 weeks compared to the initial values (Fig. 4**)**.

**Fig. 5:**
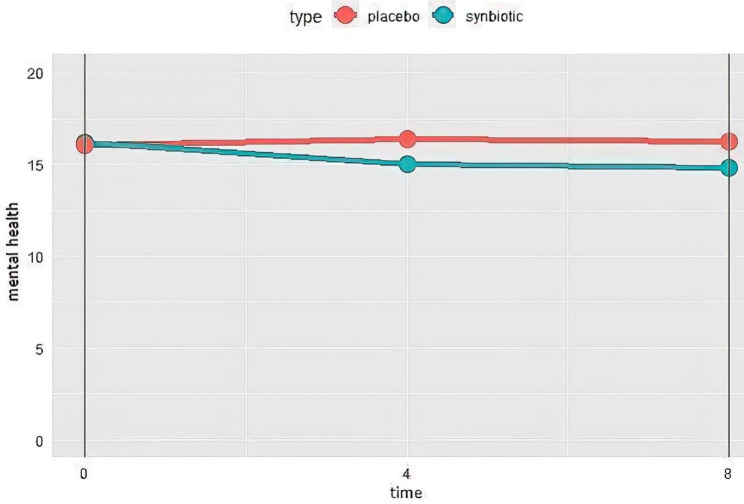
Changes of mental health in synbiotics and control groups during the study. Lower score means better mental status

There was a slight decrease in the score of the patients’ mental status in the synbiotics group during the study (improvement), whereas a high decrease was reported in the placebo group (worsening). Nonetheless, none of these changes were significant (Fig. 5**)**.

**Fig. 6:**
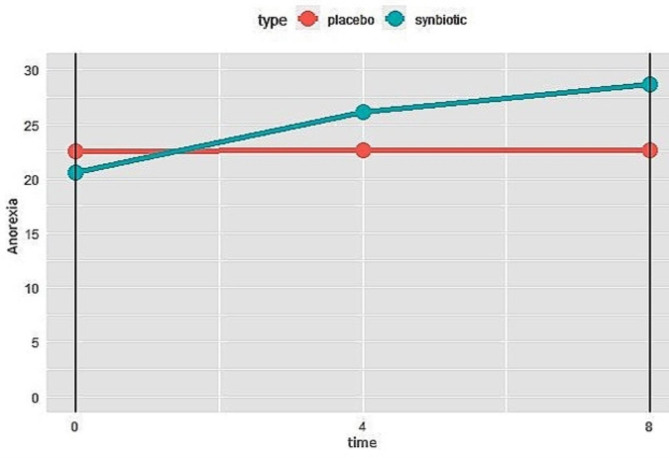
Changes of anorexia severity in synbiotics and control groups during the study. Higher score means less anorexia

According to the AC/S questionnaire score, compared to the first visit, anorexia decreased significantly at the end of the study (P < 0.001) in the synbiotics group. This was also significant at week 4 (P < 0.001). But these changes in anorexia were not statistically significant compared to the changes in the placebo group. Whereas, based on VAS score, there was a significant difference between two groups (P = 0.015), but there was no significant difference after adjusting the confounders (protein, fat, fiber, calcium and vitamin C intake) (Fig. 6**)**.

**Fig. 7:**
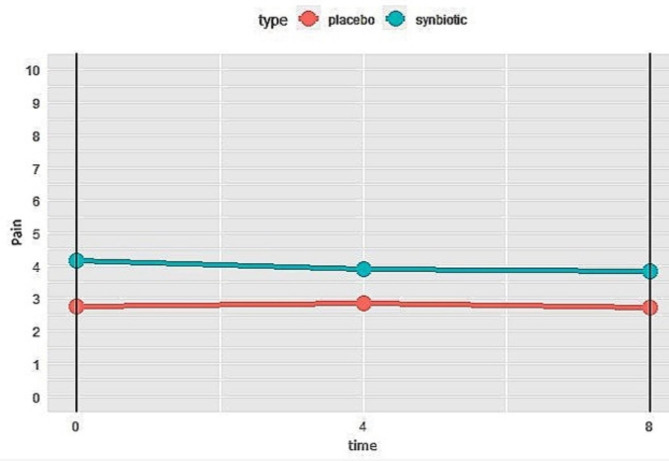
Changes of pain severity in synbiotics and control groups during the study. Higher score means more pain

Despite the reduction of pain severity in the synbiotics group during the study period, it was not statistically significant (Fig. 7**)**.

**Fig. 8:**
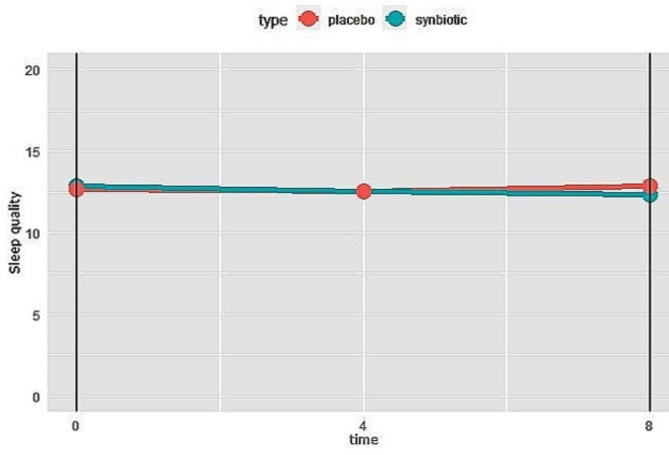
Changes of sleep quality in synbiotics and control groups during the study. Higher score means less sleep quality

The changes of sleep quality of participants are presented in Fig. 8. As it can be seen, they did not vary significantly during the study within each group. Additionally, there was no significant difference in sleep quality between the two groups at the end of the study.

Patients in the synbiotics group gained weight, while the patients in the placebo group lost weight during the study period, but the changes were not statistically significant between the two groups at the end of study and also within each group compared to the baseline.

Despite the increment of BMI in the symbiotic group and the reduction of BMI in the placebo group, there was no statistically significant difference, neither between the two groups, nor within each group compared to the baseline.

**Fig. 9:**
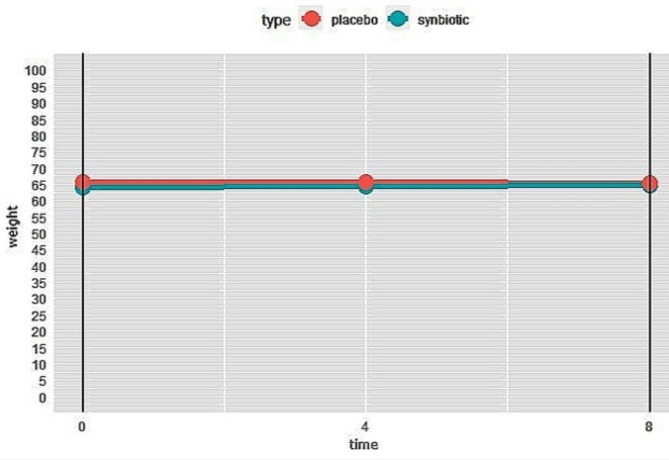
Changes of in weight in synbiotics and control groups during the study

**Fig. 10:**
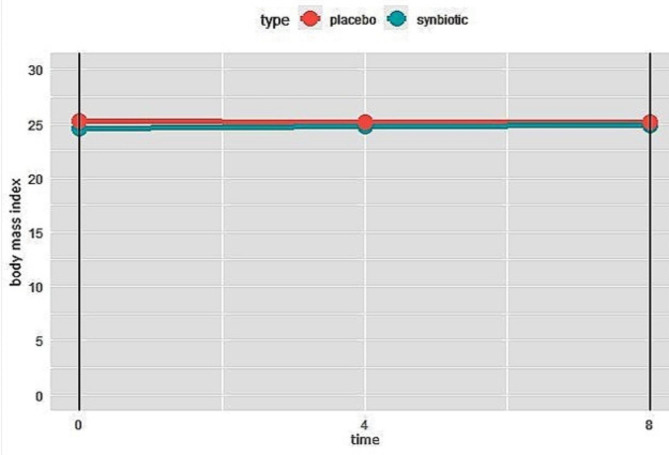
Changes of body mass index weight in synbiotics and control groups during the study

Waist circumference didn’t change significantly during the study time compared to the first visit within each group. There was no significant difference between two groups at the end of the study (Figs. 9 and 10**)**.

No signs of side effects were reported by the patients during the study.

## Discussion

To the best of our knowledge, to date, this is the first randomized clinical trial to assess the contributory effects of oral synbiotics intake on common side effects of chemotherapy, such as nausea and vomiting, defecation quality, fatigue, sleep quality, anorexia, and mental status in women with breast cancer. Due to annual increase of breast cancer around the world, and the disruptive effects of chemotherapy agents on the quality of patients’ lives as the main treatment to control breast tumors, we conducted this randomized clinical trial to realize if synbiotics therapy can effectively reduce common annoying complications of chemotherapy. So far, all studies investigating the effects of synbiotics on breast cancer have examined the paraclinical indicators or were conducted on breast cancer survivors after completing chemotherapy courses [33–35], and none of the studies have focused on the complications that patients experience especially during chemotherapy. The results indicated that the consumption of synbiotics supplements for 8 weeks can significantly improve fatigue and defecation quality in breast cancer patients with no side effects. Moreover, anorexia, nausea and vomiting were significantly reduced in the synbiotics group compared to the first visit, even though, they were not significant compared to the placebo group.

Several studies have focused on the beneficial effects of live bacteria on cancer outcomes. Likewise, in their systematic review, Eslami et al. ( 2019 ) claimed that using probiotic microorganisms can beneficially affect the management and treatment of colorectal cancer through positive alteration of intestinal microflora, fighting against inflammation, reducing cytotoxic and genotoxic agents, suppressing tumor cell proliferation, growth and metastasis, and invigorating gut permeability [36]. On the other hand, Lages et al. in their randomized clinical trial (2018 ) on 36 patients with head and neck cancers who had undergone surgery, found that the intake of synbiotics for 5 to 7 days after surgery, could not significantly alter diamine oxidase as a marker for gastrointestinal permeability [37]. This failure was attributed to the unsuitability of DAO as the best marker for gut penetrance, not the uselessness of probiotics [14]. Few studies have assessed the impact of live microorganisms on the complications of cancer treatments. According to Lu et al. ( 2019 ), only 3 studies compared the effects of probiotics on chemotherapy-induced diarrhea with placebo during treatment and one study was conducted on the prevention of diarrhea in patients with different cancers. They found that probiotics could lower the severity of diarrhea, but it is not effective in mild to moderate severity. However, the majority of studies were on colorectal cancer or leukemia, and none was on breast cancer [38]. The results of our study indicated that none of the participants in the synbiotics group reported diarrhea of any severity. None of the patients suffering from diarrhea in the synbiotics group reported this complication at first, even 4 weeks after the intervention. This is in line with the results of the study by Linn et al. (2019), who after a 3-week intervention found that the simultaneous intake of *Lactobacillus acidophilus* and *Bifidobacterium animalis*, 3 times a day, can potentially reduce the incidence and the severity of diarrhea in cervical cancer patients under radiotherapy, and reduce the need for Loprimide [39]. However, some studies do not support the effects of probiotics on diarrhea in cancer patients. Similarly, a meta-analysis in 2017 rejected the positive prophylactic effects of probiotics in reducing toxic treatment-induced diarrhea [40]. This discrepancy between non-supportive results and ours, could be due to our different supplement composition. Most of the studies assessed in this meta-analysis had applied only one or two strains, while the synbiotics supplement in this study contained 12 different bacteria and a prebiotic combined with probiotics. The better effectiveness of multiple probiotics strains is confirmed by previous evidence [41]. The underlying mechanisms of synbiotics on chemotherapy-induced diarrhea are not clear, yet. But evidence suggests that probiotics may improve diarrhea through reducing intestinal acidity, reducing pathogens proliferations, affecting immunity and lowering intestinal inflammation levels, and suppressing the apoptosis of epithelial cells as the main causes of diarrhea in invasive treatments. Moreover, probiotics protect villus against atrophy which leads to up-regulation of lactase and better lactose tolerance [39]. Furthermore, the beneficial effects of probiotics on diarrhea by protecting and improving mucosal barrier against chemotherapy agents detriments, are mentioned by current study [13].

The results of this study are consistent with those of the study by Reyna-Figueroa et al. (2019) on 60 leukemia children undergoing chemotherapy. They found that the intake of *Lactobacillus rhamnosus* ,twice a day during the entire chemotherapy period, improved constipation and nausea/vomiting as well [13]. In our study, only one patient out of 14 suffering from constipation, reported this abnormal defecation 8 weeks after symbiotic therapy. In addition, our results showed that the severity of nausea/vomiting significantly reduced at the end of the intervention period compared to the first visit. The difference between the results of the study by Reyna-Figueroa and ours, could be attributed to different dosages applied in two studies. The dosage of *Lactobacillus rhamnosus* in their study was 5 times higher than the dosage we used in our supplement. Another compatible study conducted on 100 cancer patients of various types in China (2014) showed that in addition to diarrhea, the constipation-induced chemotherapy improved 4 weeks after the consumption of *Bifidobacterium* [42]. Likewise, a study in 2018 investigated the effect of *Bacillus licheniformis* (three times a day) on consumption during the entire radiotherapy period on 160 pediatric patients with central nervous system cancer. According to the results, in addition to significant improvement in diarrhea, the severity of nausea/vomiting significantly reduced [43]. The fact that there was no significant difference between our synbiotics and placebo group regarding nausea/vomiting, could be due to the low sample size or adjusting large numbers of cofounders in statistical analysis.

Probiotics increase mucin secretion which leads to the prevention of dysmotility and modulates defecation volume and texture [13].On the other hand, they enhance the load of useful bacteria in the intestine which in turn results in higher fermentation and production of fermented short chain fatty acids, like acetic acid that cause peristalsis stimulation, better intestinal transition, and easier defecation [42]. All these can be considered as possible ameliorating effect of probiotics on constipation. In addition, prebiotics are found to be able to rise fermentable products including shore chain fatty acids, vitamin B and K and potentially boost the beneficial effects of symbiotic supplementation [44]. Following the toxic and inflammatory impacts of chemotherapy in gastrointestinal and central nervous system, these are the triggers of upper gastrointestinal tract symptoms, like nausea/vomiting.

One of the probable mechanistic pathways of synbiotics on nausea/vomiting is that probiotics decrease inflammation levels and protect immune barriers, and down-regulate the pre-inflammatory cytokines (e.g., TNF-α, IL-6, IL-8, IL-1β) that results in suppressing the toxic effects of chemotherapy drugs [43]. This mechanism is confirmed by Diop et al. who conducted a study on patients experiencing upper gastrointestinal problems. They reported that taking *Lactobacillus acidophilus* and *Bifidobacterium longum* for 3 weeks, significantly relieved stress-induced nausea/vomiting. In line with our results and all mentioned publications, also a meta-analysis on 11 randomized clinical trials in 2020 showed that using probiotics/synbiotics preoperatively as an easy and low-cost administration, in colorectal cancer surgery volunteers, had a significant beneficial effect on postoperative gastrointestinal outcomes [44]. This achievement is consistent with the results of the systematic review by Araújo et al. in 2023 [45]. However, the traditional therapeutic approach, such as using antidiarrheal or bulk-forming medication, did not have a remarkable efficacy [44].

Among non-gastrointestinal side effects, we observed a significant reduction in fatigue experience in both week 4 and week 8 in the synbiotics group. In contrary, the placebo group experienced more fatigue at the end of study. These changes were also statistically significant between the two groups and didn’t change even after adjusting the confounders. These results are in line with those of the study by Lee et al. (2014,) who reported that probiotics consumption (*L. rhamnosus and L. acidophilus*) with a dosage of 5 × 10^9^ CFU for 12 weeks could significantly reduce the severity of cancer-induced fatigue in colorectal cancer patients. Additionally, they found a significant improvement in patients’ mental health [12]. Cancer or cancer treatments-induced fatigue usually is accompanied by depression or anxiety. Dysbiosis and following Genomic and/or biochemical changes caused by chemotherapy as a toxic intervention, may trigger some brain associated interactions that are responsible for psychological problems and fatigue experience. They all refer to the gut-brain axis and the tight connection between the gut microbiome and the nervous system [12, 46, 47]. There are several studies that have confirmed the prophylactic effects of pro-/syn- biotics supplementation on non-cancerous related fatigue or psychological problems [48, 49]. Accordingly, the review article by Chudzik et al. in 2021 mentioned that pre-, pro- and post-biotics are potentially associated with better mood and lower biomarkers of depression [46], and the systematic review by Roman et al. in 2018 confirmed the potential modulator roles of probiotics on the inflammation levels and anxiety features in cancer fatigue syndrome (CFS) [49]. Although, we did not find a statistically significant reduction in changes of mental status, the reduction of PHQ-9 score in the synbiotics group (improvement), and increment of it in the placebo group (worsening) was visible. There was no significant difference in our patients’ mental status, which could be due to the small sample size and the large number of confounder variables. Furthermore, previous studies were mostly conducted in mild conditions, but patients in our study were undergoing chemotherapy, an aggressive inflammatory condition. Based on current evidence, pre, pro or synbiotics interventions are more effective in mild inflammation compared to high inflammatory situations [50]. On the other hand, the beneficial impacts of high fiber diets on psychological health and CFS are suggested in previous evidence. Increasing the load of Enterococcus and Streptococcus in normal flora can raise intestinal inflammation as well as systemic inflammation that provoke fatigue experience and severity. Thus, more intake of fermentable carbohydrates and less Firmicutes/Bacteroidetes ratio fights the inflammation- induced fatigue and when used with probiotics, can have synergic effects [51]. In line with this study, we chose synbiotics that contain both pro- and prebiotics.

In addition to the above complications, the results of our study indicated that anorexia reduced significantly at both week 4 and week 8 although the changes of anorexia were not significant between the two groups. Insufficient dietary intake and cachexia, following the loss of appetite and food aversion, is associated with lower treatment response and quality of life in these patients [52]. Anorexia in cancer patients occurs due to pre-inflammatory cytokines produced by tumor cells, changes in appetite-related hormones, disturbing of gastrointestinal homeostasis and nausea/vomiting occurrence, dysregulation of neurotransmitters or neuropeptides in appetite signaling which not only reduce the appetite, but also lead to other complications, like depression or pain [53]. The metabolic disturbance caused by anorexia and raising basal energy expenditure level in such patients lead to adipose tissue and lean mass breakdown, that eventually, end in weight loss and cachexia [54]. The relationship between anorexia and probiotics consumption has not been evaluated directly in cancer patients, but several experimental and in vitro studies have assessed appetite regulation via hormonal/neuronal signaling [55–57]. For example, a study on cachectic mice showed that intake of cancer preventive kimchi (with a standard recipe and added probiotics, could significantly down-regulate the genes related to the muscle atrophy and IL-6 levels, a potential factor related to the cachexia which reduces lipolysis, and improves the appetite [58]. Due to the mechanism of appetite improvement related to probiotics, the recent protocol by Groner in 2022 mentioned that probiotic supplementation may be effective in the amelioration of anorexia, depression, anxiety, and weight loss in anorexia nervosa patients. These positive effects can occur through gut-brain axis, and a strong association between weight control and microbiota homeostasis [59]. Also, higher neogenesis of brain cells induced by probiotics intake, called psychobiotics, is mentioned as such possible pathways [60]. The potential impact of probiotics on modifying GI function specially in the improvement of nausea/vomiting is another confirmation to better appetite and food intake in chemotherapy patients. Kazemi et al. (2020 ) suggested that the consumption of *Lactobacillus helveticus* and *Bifidobacterium longum* can significantly regulates leptin circulation, an important hormone related to appetite, improve desire to eat, and increase energy intake, in major depressed patients, but they did find any significant effect regarding prebiotics. This study mentioned *helveticus* and *longum* as the most effective strains on mood [61] We also used the same strains in our supplement composition and our results are compatible with this study. We observed that in addition to anorexia improvement, energy intake raised significantly in the synbiotics group at the end of the study, in contrast with the patients in the placebo group who experienced a significant reduction in energy intake. Weight gain and BMI improvements were also seen in the synbiotics group, although it was not statistically significant. Following cancer or cancer treatment-induced cachexia, about 45% of patient’s lost 10% of their body weight indicating that cachectic patients have a lower intestinal microbiome variety that is tightly associated with more nutritional deficiency and higher inflammatory biomarkers [62]. A study by Bowen et al. ( 2007) suggested that a 28 –day consumption of probiotics leads to a 5.3% weight loss in mice undergoing chemotherapy compared to the control groups that experienced 12.5% weight loss [63]. Furthermore, Yuan et al. ( 2015) showed that prophylactic combination of probiotics with 5-fluorouracil as the chemotherapy agent, can potentially results in a higher average of weight in mice at the end of the study compared to the placebo group [64]. A comprehensive review by Miknevicius et al. in 2021 has also confirmed the effectiveness of probiotics supplements on preventing weight loss in cancer patients [65]. In contrary, Jiang et al. in their placebo-controlled clinical trial in 2019, observed that the intake of probiotics during chemoradiotherapy of nasopharyngeal carcinoma ameliorated the oral mucositis, but couldn’t effectively prevent weight loss [66]. This discrepancy may be due to applying radiotherapy to the head and neck region in these patients that is directly related to food intake.

The improvement of diarrhea following a better intestinal absorption and lower intestinal mucositis [67], as well as constipation and fullness amelioration that cause more desire to eat, are the other reasons for lower weight loss in probiotics or synbiotics consumers. In addition, it is emphasized that probiotics intake can effectively improve the absorption of nutrients and natural microbial vitamins, better intestinal toxins exertion, and lower inflammatory cytokines related to losing weight in cancer patients [42]. Interestingly, the consumption of macro- and micronutrients increased in the synbiotics group; therefore, their protein, fat, fiber, calcium, and vitamin C intake increased compared to the placebo group. Additionally, there was a clear increment in energy intake in patients of the synbiotics group, and an obvious decrease of energy intake in the placebo group, compared to the baseline. So, this can legitimize our findings about the improvement of anorexia and better weight control in symbiotic group, compared to the placebo group. We observed some weight gain in the synbiotics group and some weight loss in the placebo group, although, none of these observations were statistically significant, which can be due to the small number of samples or the presence of many confounders. Anyway, our results are aligned with those of the study by Tian et al. ( 2019 ) who found that a 3- week consumption of *Clostridium butyricum* by lung cancer patients reduced the weight loss compared to the non-consumers, but it was not statistically significant. They mentioned short follow-up period as the main cause of nonsignificant results [68]. The findings in this study are subject to certain limitations, including the inadequate sample size (due to the time of sampling in the COVID-19 pandemics, and the concern of these patients and their families to get any extra medication during chemotherapy), and also the presence of many confounders (limited access to eligible patients meeting all inclusion criteria); therefore, we could not statistically analyze diarrhea and constipation separately and provide numbers, so we reported these two complications through descriptive analysis. One of the strengths of this study, was that no studies have comprehensively investigated the effect of synbiotics consumption on common chemotherapy-induced side effects in breast cancer patients. Moreover, the validity and reliability of all questionnaires in this study had been previously confirmed on cancer patients. In addition, lots of studies merely concentrated on the use of probiotics, while in our study we used synbiotics supplement, which is a combination of prebiotics and probiotics. In addition, most studies evaluated only one or two bacterial strains; however, we used 12 bacterial strains in our supplement composition. Also, in contrast to many clinical trials with only before and after intervention, we designed our assessment in three measurement times (beginning, week 4 and week 8 after synbiotics supplementation), and the comparison of the intervention effects between different times was also statistically investigated.

## Conclusion

Generally, our findings in this study showed that 8-week synbiotics supplementation in women with breast cancer undergoing chemotherapy could significantly reduce the severity of abnormal defecation problems and fatigue. Also, synbiotics seem to be promising for ameliorating nausea/vomiting, anorexia and weigh loss in these patients by more well-designed clinical trials with a bigger sample size, in future. In addition, it is recommended to conduct more examinations to establish a standard therapeutic protocol for the administration of synbiotics in such patients.

## Data Availability

The datasets used and/or analyzed during the current study are available from the corresponding author on reasonable request. requests should be sent to Dr. Farzad Shidfar. E-mail: shidfar.f@iums.ac.ir.

## Declarations

All methods were carried out in accordance with relevant guidelines and regulations. Also, informed consent was signed by all of included patients.

## Abbreviations

ALT: Alanine Transaminase
AST: Aspartate Transaminase
BMI: Body Mass Index
CFS: Cancer Fatigue Scale
CRP: C-Reactive Protein
CRF: Cancer Related Fatigue
CINP: Chemotherapy-Induced Peripheral Neuropathy
CNIV: Chemotherapy-Induced Nausea and Vomiting
IL-6: Interleukin-6
IRCT: Iran Registry of Clinical Trials
IPAQ: International Physical Activity Questionnaire
MET: Metabolic Equivalent
MUFA: Mono Unsaturated Fatty Acids
PHQ-9: Physical health questionnaire-9
PUFA: Poly Unsaturated Fatty Acids
SFA: Saturated Fatty Acids
VAS: Visual Analog Scale
WBC: White Blood Cells
